# Supplementary material for: Evaluating theory of change to improve the functioning of the network for improving quality of care for maternal, newborn and child health
Source: PLOS Glob Public Health. 2024 Aug 1;4(8):e0003532. doi: 10.1371/journal.pgph.0003532 (PMC11293647; doi:10.1371/journal.pgph.0003532)
Supplement: Attachment — Submitted filename: PGPH-D-23-02391 response to reviewers.docx [file pgph.0003532.s003.docx]

PGPH-D-23-02391

Evaluating theory of change to improve the functioning of The Network for Improving Quality of Care for Maternal, Newborn and Child Health

PLOS Global Public Health

Dear Editor

Thank you for providing feedback on our work and giving our team the opportunity to improve our manuscript. We would like to also thank Emma Radovich for her time reviewing our manuscript. Please find below our response to the comments received.

Best regards,

Prof. Tim Colbourn and Albert Dube, on behalf of all authors

Major feedback:

1. I understand that many details about the QCN are undoubtably reported elsewhere, but reading the paper is occasionally challenging because of lingering questions about what exactly the QCN was and what was found in the other studies of the evaluation. One or two sentences about what the QCN did (for example, what were the “learning facilities”?) and what the overall evaluation of the network found would be very helpful to understanding this study’s results. This contextual background might fit well in the paragraph beginning line 90.

Authors’ response: Thank you for highlighting this issue. We provide background on the QCN in lines 55 to 61 of our introduction [please note all line numbers refer to the Tracked changes version]: “In 2017, WHO and global partners launched ‘The Network for Improving Quality of Care for Maternal, Newborn and Child Health’ (QCN) (7) seeking to reduce in-facility maternal and newborn deaths and stillbirth by 50% in health facilities by 2022, initially in nine countries: Bangladesh, Cote d’Ivoire, Ethiopia, Ghana, India, Malawi, Nigeria, Tanzania and Uganda. Their aim was to build a cross-country platform for joint-learning around quality improvement implementation approaches based on a shared theory of change and shared health outcome goals.” To this we have added (lines 61-65­) more on what the QCN did: “The QCN operated at global, national and local levels facilitating implementation of a range of quality improvement efforts for different aspects of maternal, newborn and child healthcare, starting in selected ‘learning facilities’ in ‘learning districts’ and scaling up to other health facilities and districts (7)” We think this background information fits best in the Introduction section.

We have also added a sentence on the overall findings of our evaluation of the QCN to the first paragraph of the methods after we introduce the other papers of our QCN Evaluation collection (lines 116-119): “The main findings of our evaluation were that global and national leadership elements of QCN have been most effective to date; action, learning and accountability have been more challenging, partner or donor dependent, remaining to be scaled-up, and pandemic-disrupted.”

2. Further, is there any background information about how the international and national partners developed their theory of change and how it was intended to be used (paragraph beginning line 78)?

Authors’ response: We have added a sentence and reference 21 explaining how the theory of change was developed (lines 91-94): “The theory of change was developed by global level stakeholders of the QCN (led by the WHO and UNICEF core group) from September 2016 to February 2017 and shared with 340 national stakeholders and partners at the launch of the network in Lilongwe, Malawi in February 2017 (21).“.

References 21:	The Network for Improving Quality of Care for Maternal Newborn and Child Health. Quality, equity, dignity: the network to improve quality of care for maternal, newborn and child health – strategic objectives (<https://apps.who.int/iris/bitstream/handle/10665/272612/9789241513951-eng.pdf> accessed 20 Mar 2024). Geneva: World Health Organization; 2018.

Please see our response to point 3 below on how the theory of change was intended to be used.

3. Lines 69-75: The authors note that theories of change can have multiple applications, and the phrase is sometimes used with multiple meanings. Do the authors, or the creators of the QCN theory of change, take a particular stance on what the ‘theory of change’ is in this case?

Authors’ response: Yes, we have added what the creators of the QCN theory of change intended it to be used for, again referencing newly added reference 21 (lines 98-100): “The theory of change was used by QCN stakeholders primarily as a logical framework to guide areas of implementation, and to guide monitoring and evaluation of the network (7, 21)”. In our paper we already explain our stance, at the end of the introduction (lines 104-106): “The aim of this paper is to explore how the quality of care network theory of change guided what actually happened over 2018-2022 in order to understand what worked well, what did not, and to ultimately help explain the consequences of QCN activities”

4. Figure 1 seems scant on some details, particularly around the assumptions that seem integral to a ‘proper’ theory of change and that are of core interest in this analysis. How did the authors go about investigating assumptions that may not have been stated explicitly in this diagram?

Authors’ response: Thanks for highlighting this – this is a key issue and one of the findings of our work – that the QCN theory of change did not sufficiently explore assumptions in their theory of change (e.g. in relation to required infrastructure or data, or how often sharing and innovation would occur even with facilitation), and this could have limited how well the QCN was able to achieve things (see e.g. lines 270-273, 285-290). We investigated assumptions primarily through our qualitative interviews, but also through document review and observations, as explained in our methods section (lines 119-133).

5. One suggestion for the paper is to better connect the three components of investigation – results, assumptions, and causal pathways (lines 124-127) – to the findings presented. And given the large number of assumptions and causal pathways that underlie such a complex programme, how did the authors approach the need to prioritise these within the analysis?

Authors’ response: Thanks for this suggestion. Although our analysis drew on a lot of, primarily qualitative, data (interviews, document review, a survey, observations) we were ultimately limited by the data we had and were not able to explore all assumptions and potential causal pathways, as we acknowledge in our discussion (lines 408-418). We have added to this in response to point 6 below.
As we explain in the first paragraph of our results section, we structure our results by theory of change intervention themes, processes, and clinical and behavioural outcomes. We consider this structure to be coherent and within this we consider outcomes (results) as well as processes (pathways) and assumptions.

6. The limitations note that not all areas of the theory of change could be investigated (lines 366-367), but how might the choices made in the analysis, or gaps in the data, leave limitations in our understanding of the programme?

Authors’ response: We have added four sentences here to explain this (line 408-418): “Although our analysis drew on a lot of primarily qualitative data (interviews, document review, a survey, observations), we were ultimately limited by the data we had and were not able to explore all assumptions and potential causal pathways. Our investigation was also limited by the original theory of change not explicating assumptions and mechanisms that might have informed our evalution, and by the QCN not having any in-built evaluations at country level that themselves exposed and examined assumptions and mechanisms. This left some gaps in both our understanding, and the QCN team’s understanding, of the programme. For example, in relation to whether, how, and how much, the QCN was able to improve access to care for marginalised groups, improve dignity and satisfaction of patients, and reduce mortality and morbidity in network learning facilities or more broadly.

7. Is it possible to add the job title/category to the respondent descriptors in the attributions of the quotes? If there are concerns about confidentiality of participants, even very broad categories (‘facility staff’ or ‘sub-national partner’) would help to contextualise the quotes presented.

Authors’ response: Thank you for highlighting this issue. We have added participant category to each of the quotations that have been used in this paper.

8. Did none of the nine countries in the QCN develop country-level theories of change (or perhaps something else? impact pathways or logic models)?

Authors’ response: As far as we know, by 2022 (the end of our evaluation period) they had not. This was also raised and confirmed at the QCN meeting with representatives of all 11 countries in Accra, Ghana in March 2023.

9. Line 51: There’s a misprint here of the number of maternal deaths in 2020, by a magnitude of millions!

Authors’ response: Thank you for highlighting this issue. We have revised this to ’In 2020 over 2.4 million newborn babies and an estimated 287 000 women died’ (lines 53 and 54)

10. Lines 60-63: I strongly agree with the authors that interventions to improve quality of care “are complex and require comprehensive and detail [sic] theories of change”. However, I wonder whether it is the theory of change itself that contributes nuanced understanding for addressing evidence-to-practice gaps or is it the evaluations that are guided by these theories of change? (Good) evaluations interrogate assumptions and causal linkages embodied in theories of change, and we hope that this results in better design and implementation of programmes, which might be expressed in a more informative programme theory of change?

Authors’ response: Good point – we think it’s both, and have added a sentence here (lines 72-74): “Investigating assumptions and causal linkages set out in theories of change through evaluation can also contribute to better design and implementation of programmes, and refined theories of change.”

11. Line 99: Could the authors please provide more detail on who the “key stakeholders” included? Would these have included facility managers and/or staff on the frontlines of care? Ministry of Health officials? Others?

Authors’ response: Thank you for highlighting this issue: Yes, we have added more information about key stakeholders that participated as follows (lines 121-125): “Iterative rounds of interviews were conducted with key stakeholders such as QCN coordinators, UNICEF, WHO, UNFPA, and GIZ national representatives, Ministry of Health monitoring and evaluation officers, directors of health and social services at each learning facility, and quality improvement officers in each country at both national and local level”

12. Line 115: Could the authors please add a citation to the adapted tool? This would be useful for others interested in conducting similar surveys.

Authors’ response: The adapted tool (and the citation for the original tool) is contained within supplementary material S2_Text, which is cited at the end of this paragraph. We have added a citation to the end of this sentence: “S2_Text, Appendix 5)” (now line 142).

13. Lines 175-178: This quote is quite difficult to follow as it is not clear who “they” refers to here.

Authors’ response: Thank you for highlighting this issue: Yes, we have added context to the pronoun as follows: “Of course, they [Quality of care network activities] were there but doing it unknowingly, you would find that you are doing work very nicely but you are not aware, you are not appreciating yourself like you have done this one.” (Uganda-02 facility staff) (Line 203 to line 206)

14. Line 188: Should this be sub-national rather than national level initiative?

Authors’ response: Thank you for highlighting this issue: We have added more details to clarify our finding on perception of QCN activities as follows (lines 216-220): “We found that, much as the network was a government initiative within each country, funding and technical support for QCN activities was provided to national level QCN implementers by different national level NGO implementing partners. This resulted in some sub-national level actors perceiving QCN activities of the network as partner’s work rather than looking at the QCN as a government-driven initiative.”

15. Lines 351-354: The first sentence here does not seem to follow with the rest of the paragraph, and it’s not clear how these points connect.

Authors’ response: Thank you for highlighting this issue: We have added more details to clarify this as follows (lines 384-403 – please see the whole revised paragraph): “It has been argued that many of the assumptions about how the world works are based on implicit theories of change, based on our worldview, developed through our education and upbringing (38). This means that notwithstanding differences in worldviews, education levels and upbringing, differences in approaches to solving social challenges also prevail. These differences are external and outside the direct control of project management and implementers, but nevertheless should be addressed when developing programme theories of change (39). We found that key conditions were missing at service integration and process levels in the global theory of change for the network. These missing elements suggest that national level differences were either remote to the developers of the theory of change or these country level differences could not all be used to inform its development. Our findings raise questions about how a high-level, global theory of change should inform, or can inform, national or sub-regional programmes. For example, conditions such as lack of physical resources that are locality dependent were frequently reported to be preventing adequate care, or harm patient satisfaction. Lack of physical resources tied the hands of trained service providers from exercising skills and knowledge they acquired through various quality of care training sessions. We also found that some pillars of the QCN theory of change, such as community empowerment, were rarely being worked on or at least not reported on, suggesting that such pillars were not seen as a priority in the local context.”

16. Line 362: Is it that community empowerment was not applicable or simply less of a priority?

Authors’ response: Our understanding is that community empowerment was simply not prioritised rather than not being applicable. We have revised this in lines 402-403 as per above.

17. Line 373: To what extent does not reported on = absent from activities?

Authors’ response: We understand that those activities that were not being reported were not being done. We have added the following to clarify (lines 424-428): “Our review of implementation and coordination meeting reports, analysis of learning facilities observation reports, analysis of transcripts from in-depth interviews with health workers, QCN district and national level coordinators as well as international stakeholders indicated non-reporting of activities on certain pillars meant such activities were not being carried out.”
