# Supplementary material for: Evaluating theory of change to improve the functioning of the network for improving quality of care for maternal, newborn and child health
Source: PLOS Glob Public Health. 2024 Aug 1;4(8):e0003532. doi: 10.1371/journal.pgph.0003532 (PMC11293647; doi:10.1371/journal.pgph.0003532)
Supplement: Attachment — Submitted filename: Paper 5 response to re-review.docx [file pgph.0003532.s004.docx]

PGPH-D-23-02391R1
Evaluating theory of change to improve the functioning of the network for improving quality of care for maternal, newborn and child health
PLOS Global Public Health

Journal Requirements:

1. Please review your reference list to ensure that it is complete and correct. If you have cited papers that have been retracted, please include the rationale for doing so in the manuscript text, or remove these references and replace them with relevant current references. Any changes to the reference list should be mentioned in the rebuttal letter that accompanies your revised manuscript. If you need to cite a retracted article, indicate the article’s retracted status in the References list and also include a citation and full reference for the retraction notice.

Author’s response: we have checked the references and none are from retracted papers. During our checking of our references we noted we were still citing preprints of now published papers so have updated those references (24-27) and we also noted reference 31 was a duplicate of reference 23 so have deleted that.

2. We noticed that you used "unpublished" in the manuscript. We do not allow these references, as the PLOS data access policy requires that all data be either published with the manuscript or made available in a publicly accessible database. Please amend the supplementary material to include the referenced data or remove the references.

Author’s response: we don’t include unpublished data. We do state in the manuscript that we reviewed “unpublished documents” as part of our document review process (lines “121-123: “We reviewed accessible published and unpublished documents and communications relating to the quality of care network at global level and at national and local levels in each of the four countries.”). These are mainly strategy and other internal government documents from our case study countries, and are described in S2_Text (section 3.2 and Table 2 of S2_Text). We do not have the authority or permission to make these unpublished documents available. We hope this is ok and is not an issue given we only used these unpublished documents for triangulation. Kindly please also note that S2_Text is common to other papers in this collection, 5 of which have already been published by PLOS Global Health (Tesfa et al 2023, Lemma et al 2023, Mukinda et al 2023, Akter et al 2023, Djellouli et al 2024).

Reviewer #2: Firstly, I would like to congratulate the authors for the beautiful research carried out. How important and indispensable it is for us to know the reality of care for the maternal and child segment, who are so fragile in relation to health care, especially in regions of such vulnerability. I also congratulate the authors for the brilliant initiative in evaluating whether the Network and the WHO proposal actually reached the places and people who really need it.
I am very pleased to participate in this stage and, in advance, know the results in countries that require attention across the globe.

Author’s response: Thank you very much, and we are glad you appreciate our work.

My greatest concern is that, due to the magnitude of the work, the difficulties in presenting so much important data were perceived, and I fear that this may be perceived by readers as well. All the complementary material was extremely relevant for understanding the research, but I also understand when the authors mention the need for these materials to avoid overlapping information, since the entire set of results will be shown from 9 scientific articles. But this is a concern.

Author’s response: we appreciate your concern though hope the supplementary material S1_Text which provides a two-page overview of our QCN Evaluation collection of papers clarifies the breadth of our research and signposts readers to the different papers, what they cover, and how they relate to each other. We are also drafting a blog for the journal about our collection of papers, which we hope will make our research further accessible to readers.

The introduction is concise and sufficient, and shows that there are problems within the scope of maternal and child health that require resolution, and that the proposed Network is an important step, but that the theory of change needs to be evaluated as well, to understand what worked and what didn't work, what are the challenges, needs, strategies for each scenario, propose and execute these changes and achieve quality and efficiency. So I have no suggestions.

Author’s response: thank you.

Objective, clear and meets the research proposal.

Author’s response: thank you.

Regarding the method, it is partially described, with the need to consult all the supporting material to understand how the research was conducted. This will need to be made very clear to the reader. And I ask, will all this material be available for consultation?

Author’s response: yes, all of the details on our methods contained in supplementary material S2_Text will be freely available as the supplementary material will be free to read and download alongside the paper. In our paper we explain this in lines 139-140: “A detailed report on the methodology of the whole quality of care network evaluation is presented in S2 Text”.

What caught my attention was how the data collection was organized, who the researchers were, the number of researchers, how they made the first contact, what were the difficulties in accessing the participants and the documentary information, etc. This information was not described, not even in the supplementary material.

Authors response: thank you for raising these important issues. These issues are covered in another paper of our series by Gloria Seruwagi and colleagues, where we reflect on our experiences of evaluating the quality of care network. This paper, titled “How to evaluate a multi-country implementation-focused network: Reflections from the Quality of Care Network (QCN) evaluation” has just been accepted for publication in PLOS Global Public Heath (PGPH-D-23-00381R4). We have added reference to this paper on lines 141-142: “and we provide reflections on our experiences of evaluating the quality of care network in another paper in our QCN Evaluation collection by Gloria Seruwagi and colleagues (30).”

There were exclusion criteria for participants. Why were a greater number of interviews conducted in Bangladesh compared to other countries? At national and regional level? Since at the local level the number of interviews was equitable. Was there team training to conduct the interviews? What about recording all this material? How were the interviews and documents carried out?

Authors response: thank you for raising these important issues. These issues are also covered in the paper of our series by Gloria Seruwagi and colleagues, where we reflect on our experiences of evaluating the quality of care network.

There number of interviews in each country was guided by the local research teams in each country in relation to the structure of the network in each country and the need to interview the range of stakeholders in each country required to find out enough information relating to each of the aspects of the network in each country.

Details of how the interviews and document reviews were carried out is included in our supplementary material S2_Text which explains our methods.

The results were clearly described, with representations in participants' statements and a representative scheme of the main findings in each country.

Author’s response: thank you.

The limitations of the study were exposed in a timely manner in the discussion, since the impossibility of showing concrete data, in terms of access, interventions, reduction or not of maternal and child morbidity and mortality could provide more accurate data on the practical application of the network and the proposed changes . And mainly whether or not the theory of change guided health actions in the countries involved. I congratulate the authors for raising this issue at the end of the article.

Author’s response: thank you very much.

I only suggest that the authors reorganize the methodology, in order to provide more clarity to the reader on the way the study was conducted, without needing to consult all the complementary material.

Author’s response: please see our responses to your comments on this above. Our methodology is extensive so requires S2_Text to fully explain. Though as noted above, S2_Text will be freely available for readers as it will be published alongside this article were it to be accepted. We provide reflections on the way the study was conducted in a separate paper in our collection of papers on the QCN Evaluation, by Gloria Seruwagi and colleagues, which we now reference in this paper (line 142).
